# Relative Handgrip Strength Is Inversely Associated with Metabolic Profile and Metabolic Disease in the General Population in China

**DOI:** 10.3389/fphys.2018.00059

**Published:** 2018-02-05

**Authors:** Dongxue Li, Guanghong Guo, Lili Xia, Xinghua Yang, Biao Zhang, Feng Liu, Jingang Ma, Zhiping Hu, Yajun Li, Wei Li, Jiajia Jiang, Herbert Gaisano, Guangliang Shan, Yan He

**Affiliations:** ^1^Department of Epidemiology and Biostatistics, School of Public Health, Capital Medical University, Beijing, China; ^2^Department of Clinical Biochemistry, Chinese PLA General Hospital, Beijing, China; ^3^Department of Epidemiology and Biostatistics, Institute of Basic Medical Sciences, Chinese Academy of Medical Sciences, Beijing, China; ^4^Shanxi Provincial Disease Prevention and Control Center, Xi'an, China; ^5^Departments of Medicine and Physiology, University of Toronto, Toronto, ON, Canada; ^6^Municipal Key Laboratory of Clinical Epidemiology, Beijing, China

**Keywords:** handgrip strength, metabolism, association, type 2 diabetes mellitus, hyperlipidemia

## Abstract

**Background:** Absolute handgrip strength has been correlated with metabolic profile and metabolic disease. Whether relative handgrip strength is also associated with metabolic disease has not been assessed. This study aimed at assessing the association of relative handgrip strength with metabolic profile and metabolic disease in the general population in China.

**Methods:** Data were derived from an ongoing cross-sectional survey of the 2013 National Physical and Health in Shanxi Province, which involved 5520 participants. Multiple linear regression or multiple logistic regression analysis were used to assess the association of absolute/relative handgrip strength with the metabolic profile, preclinical, and established stages of metabolic diseases.

**Results:** This study revealed that relative handgrip strength, that is when normalized to BMI, was associated with: (1) in both genders for more favorable blood lipid levels of high-density lipoprotein cholesterol [males: *b* = 0.19 (0.15, 0.23); females: *b* = 0.22 (0.17, 0.28)], low-density lipoprotein cholesterol [males: *b* = −0.14 (−0.23, −0.05); females: *b* = −0.19 (−0.31, −0.18)], triglycerides [males: *b* = −0.58 (−0.74, −0.43); females: *b* = −0.55 (−0.74, −0.36)] and total cholesterol [males: *b* = −0.20 (−0.31, −0.10); females: *b* = −0.19 (−0.32, −0.06)]; and better serum glucose levels in males [*b* = −0.30 (−0.46, −0.15)]. (2) lower risk of impaired fasting glucose in males {third quartile [OR = 0.66 (0.45–0.95)] and fourth quartile [OR = 0.46 (0.30–0.71)] vs. first quartile} and dyslipidemia in both genders {third quartile [males: OR = 0.65 (0.48–0.87); females: OR = 0.68 (0.53–0.86)] and fourth quartile [males: OR = 0.47 (0.35–0.64); females: OR = 0.47(0.36–0.61)] vs. first quartile}. (3) lower risk of hyperlipidemia in both genders third quartile [males: OR = 0.66 (0.50–0.87); females: OR = 0.57 (0.43–0.75)] and fourth quartile [males: OR = 0.35 (0.26–0.47); females: OR = 0.51 (0.38–0.70)] vs. first quartile. However, contrary to relative handgrip strength, higher absolute handgrip strength was associated with unfavorable metabolic profiles and higher risk of metabolic diseases. These paradoxical associations were retained even after adjusted for BMI by employed a multivariate analysis.

**Conclusion:** We conclude that measurement of relative handgrip strength can be used as a reasonable clinical predictor of metabolic health and disease.

## Introduction

Grip strength is a simple, quick, and inexpensive method to measure muscle strength (Mearns, 2015), and which has been shown to be areliable predictor for future disability, frailty, metabolic syndrome, diabetes mellitus (Ortega et al., 2005; Ling et al., 2010; Chen et al., 2012; Sayer and Kirkwood, 2015; Dong et al., 2016; Nofuji et al., 2016). Absolute handgrip strength represents the strength of small muscle group in upper body when performed in a seated position; and when performed in the standing position, represents the lower arm, leg, and core muscle strengths (Lawman et al., 2016).

There are also evidences (Sayer et al., 2007; Silventoinen et al., 2009) that absolute handgrip strength is associated with metabolic profile and metabolic disease. Several studies indicated that absolute handgrip strength was associated with prediabetes among normal-weight Americans, South Asians, and elderly Chinese (Zhang et al., 2014; Mainous et al., 2016; Ntuk et al., 2017). However, other studies showed contradictory results (Fowles et al., 2014; Liu et al., 2014). A cohort study of 142,681 participants found no association between absolute handgrip strength and incidental diabetes mellitus during a follow-up of 4 years (Leong et al., 2015). Recent studies from Switzerland showed that absolute handgrip strength in adults to be only moderately or not associated with cardiovascular risk markers (Gubelmann et al., 2016, 2017). One study reported the association between cardiometabolic risk and absolute handgrip strength only existed in Korean men (not women; Yang et al., 2012), whereas another study indicated an association only in Japanese women (not men; Aoyama et al., 2011).

Since the absolute handgrip strength was closely related to body mass, using absolute handgrip strength as an indicator of muscle strength without correction for body mass, may be a contributing cause of the above conflicting results (Fowles et al., 2014; Keevil and Khaw, 2014). More recent studies have used the relative handgrip strength, which is calculated as absolute handgrip strength divided by the body mass index (BMI; Lawman et al., 2016). Using this newer standardized measurement, several studies have determined that relative handgrip strength was negatively associated with cardiometabolic risk, including the metabolic profile of fasting glucose, HDL cholesterol, and triglyceride. A population based study performed in Taiwan demonstrated that relative handgrip strength to be an indicator of cardiometabolic risk of triglyceride, total cholesterol to high density cholesterol (HDL-C) ratio, fasting glucose among middle-age and elderly persons (Lee et al., 2016). The association between relative handgrip strength and metabolic profile was also reported by a United States study of the National Health and Nutrition Examination Survey (NHANES; Lawman et al., 2016). However, there is no study to examine the association of relative handgrip strength with metabolic diseases. We hypothesized that relative handgrip strength might be also associated with metabolic disaeses through related to metabolic profiles. This study aimed to asssess the association between relative handgrip strength and metabolic diseases, and also compared the strength of association between relative handgrip strength and absolute handgrip strength with metaboic profile, preclinical, and established stages of metabolic diseases.

## Methods

### Study population

A national physical fitness and health survey was conducted from 2013 onwards covering five provinces, including Hainan, Shanxi, Qinghai, Gansu, and Jiangxi. Multi-stage, stratified, and cluster random sampling method was used for the sampling. Additional sampling details are available elsewhere (Xia et al., 2017). The Institute of Basic Medical Sciences, Chinese Academy of Medical Sciences (approval number 029-2013) approved this protocol, and informed consent was obtained from each subject.

The data for this particular study was collected from the site of the Shanxi province. For this study, the exclusion criteria included: (1) Subjects <18 years old; (2) Subjects who had suffered recent injuries, pain, inflammation, hand, or wrist surgery; (3) Subjects without complete information to define the metabolic diseases. Finally, a total of 5,520 (2,289 males, 3,231 females) aged 18–79 years subjects were included in this study.

### Data collection

The questionnaire included demographic data, smoking, alcohol drinking, and educational status of participants. Smoking status was categorized as never smokers, former smokers (stopped smoking for more than 12 months), and current smokers. Those with alcohol drinking habits were divided into never drinkers, former drinkers (stopped drinking for more than 12 months), and current drinkers. Educational level was divided into three categories: pre high school level, high school level, and post high school (Bai et al., 2016).

Anthropometric measurements included body weight, body height, waist circumference, and hip circumference, which were collected using standard methods (Klipstein-Grobusch et al., 1997). BMI was calculated as body weight in kilograms divided by body height in meter square. Absolute handgrip strength was measured using a hand-held Takei dynamometer (Takei Scientific Instruments Co. Ltd., Niigata, Japan) in a standing position with both arms naturally drooping. Before the test, the subjects could adjust the grip distance to the appropriate scale according to the size of their own hands. After completing a practice trial with both left and right single hands used singly the dominant hand was tested twice. The participants were asked to squeeze the dynamometer with the maximum force during the test with the dominant hand, exhaling during the squeeze, and rest 1 min between the two test intervals. The higher reading of the dominant hand two measurements of recorded as the absolute handgrip strength, expressed in kilograms. Participants were divided into four groups (Q1-Q4) by quadrisection according to relative handgrip strength.

The participants were restricted from doing any strenuous exercise and should have an 8–12 h of fasting before the drawing of blood to determine the metabolic profile. All blood samples were analyzed with strict quality control in a national central laboratory Chinese PLA General Hospitalin Beijing using the Olympus AU2700 (Olympus Instruments Inc., Tokyo, Japan). The assays to determine metabolic profile included: fasting glucose, serum triglyceride (TG) and total cholesterol (TC) measured respectively by the hexokinase, glycerol lipase oxidase (GPO-POD), and cholesterol oxidation methods. Low density lipoprotein (LDL-C) and high density lipoprotein cholesterol (HDL-C) was determined enzymatically (Kyowa Medex Co. Ltd., Tokyo, Japan).

### Definitions of metabolic disease

According to the American Diabetes Association (ADA; American Diabetes, 2017), impaired fasting plasma glucose (IFG) was defined as when fasting plasma glucose is 5.6–6.9 mmol/L. Diabetes was defined by a fasting glucose ≥7.0 mmol/L and/or presence of treatment with antidiabetic drug(s). According to Chinese guidelines on prevention and treatment of dyslipidemia in adults, dyslipidemia was defined as using anti-lipidemic medication(s) or having at least one of the following: TC: 5.18–6.19 mmol/L, TG: 1.70–2.15 mmol/L, LDL-C: 3.38–4.13 mmol/L and HDL-C: <1.04 mmol/L. Hyperlipidemia (Hu and Ding, 2008), which is the next stage of dyslipidemia, was defined as using an anti-lipidemia medication(s) or having at least one of the following: TC: ≥6.20 mmol/L, TG: ≥2.16 mmol/L, LDL-C: ≥4.14 mmol/L.

### Statistical analysis

All statistical analyses were conducted using the SPSS software version 23.0 for Windows (SPSS Inc., Chicago, IL, USA); and the graphs were created using the Survey Package in R, version 3.0.4. The continuous variables was expressed as mean ± *SD* and compared by Independent *t*-test. Categorical variables was expressed as numbers and percentages and compared by Chi-square test. The distribution of the continuous variables were examined by Kolmogorov–Smirnov test. For variable of TG was skewed, a log-transformation was performed. Because different measure units exist in absolute handgrip strength and relative handgrip strength, standardizing both measurements by their standard deviation would help to compare the magnitude of effects of these two measures and assist the interpretation of regression coefficient. Absolute handgrip strength and relative handgrip strength were transformed to x′ = (x–mean)/*SD* to standardize b (Regression Coefficient) and OR (odds ratio). Pearson correlation and multiple linear regression were used to assess the association between absolute/relative handgrip strength and the metabolic profile. Association of absolute/relative handgrip strength with preclinical and established stages of metabolic diseases was evaluated by an unconditional logistic regression analysis, in which age, educational, smoking, and alcohol drinking status were adjusted. In the above analysis, OR (or b) was used to assess the strength of association of absolute/relative handgrip strength with preclinical and established stages of metabolic disease (or metabolic profiles). All tests were two-sided, and a value of *P* < 0.05 was considered as significant.

## Results

### Characteristics of participants

The 5,520 subjects that were included in the study had a mean age of 46.7 ± 14.3 years, with 41.5% males (*n* = 2,289) and 58.5% females (*n* = 3,231).Mean absolute handgrip strength (HGS) was 36.3 ± 7.4 kg for males and 21.2 ± 4.9 kg for females; and corresponding mean relative handgrip strengths was 1.5 ± 0.4 and 0.9 ± 0.3 kg/BMI, respectively. Since some characteristics are differently distributed in males and females (Table 1), we stratified the participants into male and female groups in the following analyses.

**Table 1:** Characteristics of participants.

	**All (*N* = 5520)**	**Male (*N* = 2289)**	**Female (*N* = 3231)**	***P***
Age, years	46.7 ± 14.3	47.3 ± 14.3	46.2 ± 13.9	0.003
Race Han, *n* (%)	5,493 (99.5)	2,277 (99.5)	3,216 (99.5)	0.75
Educational level, *n* (%)				<0.001
Low	864 (15.7)	243 (10.6)	622 (19.3)	
Middle	3,180 (57.6)	1,391 (60.8)	1,789 (55.4)	
High	1,476 (26.7)	656 (28.7)	820 (25.4)	
Smoking, *n* (%)				<0.001
Never	3,954 (71.6)	755 (33.0)	3,199 (99.0)	
Former	266 (4.8)	260 (11.4)	6 (0.2)	
Current	1,300 (23.6)	1,274 (55.7)	26 (0.8)	
Drinking, *n* (%)				<0.001
Never	4,178 (75.7)	1,097 (47.9)	3,081 (95.4)	
Former	124 (2.2)	118 (5.2)	6 (0.2)	
Current	1,218 (22.1)	1,074 (46.9)	144 (4.5)	
Body mass index, kg/m^2^	23.6 ± 3.5	23.9 ± 3.5	23.3 ± 3.6	<0.001
Waist circumference, cm	84.4 ± 10.8	88.0 ± 10.1	82.0 ± 10.6	<0.001
Hip circumference, cm	95.7 ± 6.3	97.5 ± 5.8	94.6 ± 6.4	<0.001
GLU, mmol/L	5.2 ± 1.2	5.3 ± 1.2	5.2 ± 1.2	0.001
TC, mmol/L	4.4 ± 0.9	4.3 ± 0.9	4.4 ± 1.0	<0.001
TG, mmol/L	1.6 ± 1.3	1.8 ± 1.2	1.6 ± 1.3	<0.001
HDLC, mmol/L	1.3 ± 0.3	1.2 ± 0.3	1.4 ± 0.3	<0.001
LDLC, mmol/L	2.5 ± 0.8	2.5 ± 0.7	2.5 ± 0.8	0.29
Absolute grip strength, kg	27.4 ± 9.6	36.3 ± 7.4	21.2 ± 4.9	<0.001
Relative handgrip strength, kg/BMI	1.2 ± 0.4	1.5 ± 0.4	0.9 ± 0.3	<0.001
IFG, *n* (%)	699 (13.2)	327 (15.1)	372 (11.9)	0.001
Dyslipidemia, *n* (%)	3,089 (56.0)	1,193 (52.1)	1,896 (58.7)	<0.001
Diabetes, *n* (%)	233 (4.2)	117 (5.1)	116 (3.6)	0.006
Hyperlipidemia, *n* (%)	1,227 (22.2)	605 (26.4)	622 (19.3)	<0.001

*Data are expressed as mean ± SD or number (percentage). TC: total cholesterol, TG: triglyceride, HDL-C: high density lipoprotein cholesterol, LDL-C: low density lipoprotein cholesterol, GLU: serum glucose. P values were from Chi-square or Student's t-tests comparing males and females*.

### Higher relative handgrip strength was associated with a lower risk of preclinical and established stages of metabolic disease

Table 2 shows that the third and fourth quartiles of relative handgrip strength was significantly associated with a lower risk of a preclinical stage of metabolic disease including IFG in males [all *P* < 0.05,Q3: OR = 0.66 (CI: 0.45–0.95); Q4:OR = 0.46 (CI: 0.30–0.71)] and dyslipidemia in both genders [all *P* < 0.05, males: Q3:OR = 0.65 (CI: 0.48–0.87), Q4:OR = 0.47 (CI: 0.35–0.64); females: Q3: OR = 0.68 (CI: 0.53–0.86), Q4: OR = 0.47 (CI: 0.36–0.61)] when compared with Q1. Table 3 shows the association between relative handgrip strength and established stages of metabolic disease. The association between relative handgrip strength and diabetes was not statistically significant in both genders. The higher relative handgrip strength was significantly associated with lower risk of hyperlipidemia [all *P* < 0.05, males: Q3:OR = 0.66 (CI: 0.50–0.87); Q4:OR = 0.35 (CI: 0.26–0.47); females: Q3:OR = 0.57(CI: 0.43–0.75), Q4:OR = 0.51 (CI: 0.38–0.70)] in both genders compared to the first quartile of relative handgrip strength.

**Table 2:** Association of relative handgrip strength with preclinical stages of metabolic disease.

		**IFG**			**Dyslipidemia**		
**RHGS**	***N***	**Events, *n* (%)**	**OR (95% CI)**	***P***	**Events, *n* (%)**	**OR (95% CI)**	***P***
**MALE**							
Q1 (≤1.31)	576	111 (19.3)	Reference		337 (58.5)	Reference	
Q2 (1.32–1.52)	534	95 (17.8)	0.96 (0.69–1.34)	0.82	322 (60.3)	0.95 (0.71–1.27)	0.74
Q3 (1.53–1.76)	537	66 (12.3)	0.66 (0.45–0.95)	0.027	269 (50.1)	0.65 (0.48–0.87)	0.004
Q4 (≥1.77)	571	43 (7.5)	0.46 (0.30–0.71)	<0.001	222 (38.9)	0.47 (0.35–0.64)	<0.001
**FEMALE**							
Q1 (≤0.74)	780	144 (18.6)	Reference		551 (70.6)	Reference	
Q2 (0.75–0.92)	740	101 (13.7)	0.99 (0.74–1.33)	0.96	478 (64.6)	0.87 (0.68–1.12)	0.28
Q3 (0.93–1.09)	771	68 (8.8)	0.77 (0.55–1.08)	0.13	427 (55.4)	0.68 (0.53–0.86)	0.002
Q4 (≥1.10)	794	46 (5.8)	0.72 (0.48–1.06)	0.10	347 (43.7)	0.47 (0.36–0.61)	<0.001

*CI, confidence interval; RHGS, relative hand grip strength. P-values were from logistic regression, in which age, sex, smoking, alcohol drinking, and educational status was adjusted*.

**Table 3:** Association of relative handgrip strength with established stages of metabolic disease.

**RHGS**		**Diabetes**			**Hyperlipidemia**		
	***N***	**Events, *n* (%)**	**OR (95%CI)**	***P***	**Events, *n* (%)**	**OR (95% CI)**	***P***
**MALE**							
Q1 (≤1.31)	576	44 (7.6)	Reference		185 (32.1)	reference	
Q2 (1.32–1.52)	534	30 (5.6)	0.99 (0.60–1.65)	0.99	162 (30.3)	0.86 (0.66–1.11)	0.25
Q3 (1.53–1.76)	537	25 (4.7)	1.02 (0.59–1.76)	0.94	139 (25.9)	0.66 (0.50–0.87)	0.003
Q4 (≥1.77)	571	15 (2.6)	0.82 (0.43–1.57)	0.55	91 (15.9)	0.35 (0.26–0.47)	<0.001
**FEMALE**							
Q1 (≤0.74)	780	54 (6.9)	Reference		243 (31.2)	Reference	
Q2 (0.75–0.91)	740	30 (4.1)	0.90 (0.56–1.45)	0.66	166 (22.4)	0.84 (0.66–1.07)	0.16
Q3 (0.92–1.09)	771	23 (3.0)	0.94 (0.55–1.62)	0.83	108 (14.0)	0.57 (0.43–0.75)	<0.001
Q4 (≥1.10)	794	5 (0.6)	0.33 (0.12–0.88)	–	78 (9.8)	0.51 (0.38–0.70)	<0.001

*CI, confidence interval; RHGS, relative hand grip strength P-values were from logistic regression, in which age, sex, smoking, alcohol drinking, and educational status was adjusted. –, The subjects with diabetes in the fourth quartile of relative handgrip strength is too few (only five) in females, to avoid affecting the stability of the model, which isn't included in the analysis*.

### Higher relative handgrip strength was associated with a favorable metabolic profile

To assess whether relative handgrip strength was associated with preclinical and established stages of metabolic diseases was related to its influence on metabolic profiles, we further examine the association of relative handgrip strength with metabolic profiles. For males, relative handgrip strength ranged from 0.43 to 2.79 kg/BMI with interquartile range (IQR) of 0.45 kg/BMI. For females, relative handgrip strength ranged from 0.21 to 1.9 kg/BMI with IQR of 0.36 kg/BMI. Higher relative handgrip strength was significantly associated with favorable LDL-C [males: *P* = 0.003, *b* = −0.14 (CI: −0.23, −0.05); females: *P* = 0.001, *b* = −0.19(CI: −0.31, −0.18)],TC [males: *P* < 0.001, *b* = −0.20 (CI: −0.31, −0.10); females: *P* = 0.005, *b* = −0.19 (CI: −0.32, −0.06)], TG [all *P* < 0.001, males: *b* = −0.58(CI: −0.74, −0.43); females: *b* = −0.55 (CI: −0.74, −0.36)] in both genders, GLU [*P* < 0.001, *b* = −0.30 (CI: −0.46, −0.15)] in males and HDL-C [all *P* < 0.001, males: *b* = 0.19 (CI: 0.15, 0.23); females: *b* = 0.22 (CI: 0.17, 0.28)] in both genders (Table 4).

**Table 4:** Association between relative handgrip strength and metabolic profile.

	**Male (*****n*** = **2,289)**				**Female (*****n*** = **3,231)**			
	***P*^1^**	***r***	***P*^2^-value**	**B (95%CI)**	***P*^1^**	***r***	***P*^2^**	**B (95%CI)**
GLU	<0.001	−0.165	<0.001	−0.30 (−0.46 to −0.15)	<0.001	−0.155	0.12	−0.16 (−0.32 to 0.04)
HDL-C	<0.001	0.162	<0.001	0.19 (0.15 to 0.23)	<0.001	0.167	<0.001	0.22 (0.17 to 0.28)
LDL-C	<0.001	−0.133	0.003	−0.14 (−0.23 to −0.05)	<0.001	−0.202	0.001	−0.19 (−0.31 to −0.08)
TC	<0.001	−0.138	<0.001	−0.20 (−0.31 to −0.10)	<0.001	−0.226	0.005	−0.19 (−0.32 to −0.06)
TG^*^	<0.001	−0.182	<0.001	−0.58 (−0.74 to −0.43)	<0.001	−0.325	<0.001	−0.55 (−0.74 to −0.36)

GLU, serum glucose; HDL-C, high density lipoprotein cholesterol; LDL-C, low density lipoprotein cholesterol; TC, total cholesterol; TG^*^, means of log-transformed for triglyceride.P^1^-values were from Pearson correlation. P^2^-values were from multivariable linear regression, in which age, sex, smoking, alcohol drinking, and educational status was adjusted.

### Relative handgrip strength was a better predictor for metabolic disease than absolute handgrip strength

**Figure 1:**
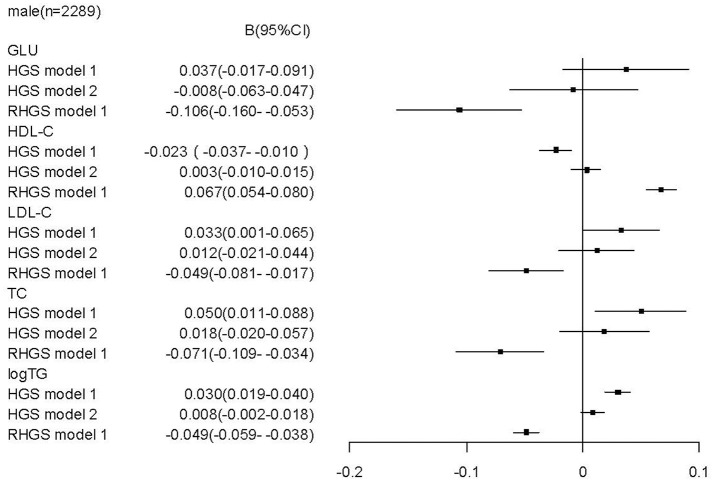
Association of absolute handgrip strength, relative handgrip strength with the metabolic profile in male subjects.TC, total cholesterol; logTG, means of log-transformed for triglyceride; HDL-C, high density lipoprotein cholesterol; LDL-C, low density lipoprotein cholesterol; GLU, serum glucose; HGS, absolute handgrip strength; RHGS, relative handgrip strength. Absolute handgrip strength and relative handgrip strength were standardized before entering into the linear regression model. Model 1: adjusted for age, smoking, alcohol drinking, and educational status; Model 2: Model 1+ adjusted for BMI.

**Figure 2:**
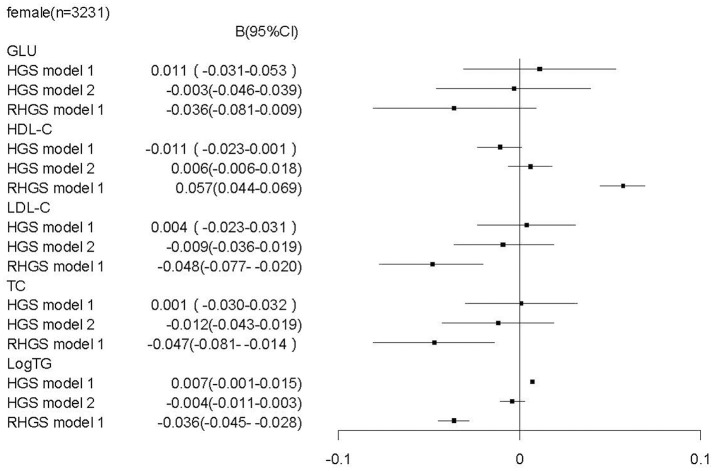
Association of absolute handgrip strength, relative handgrip strength with the metabolic profile in female subjects.TC, total cholesterol; logTG, means of log-transformed for triglyceride; HDL-C, high density lipoprotein cholesterol; LDL-C, low density lipoprotein cholesterol; GLU, serum glucose; HGS, absolute handgrip strength; RHGS, relative handgrip strength. Absolute handgrip strength and relative handgrip strength were standardized before entering into the linear regression model. Model 1: adjusted for age, smoking, alcohol drinking, and educational status; Model 2: Model 1+ adjusted for BMI.

**Figure 3:**
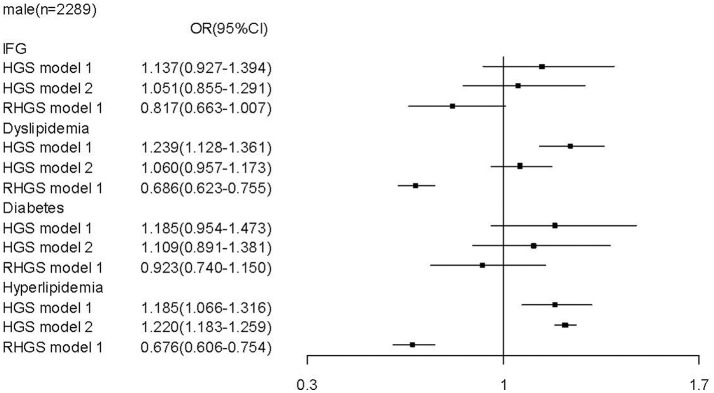
Association of absolute handgrip strength, relative handgrip strength with metabolic disease in male subjects.TC, total cholesterol; logTG, means of log-transformed for triglyceride; HDL-C, high density lipoprotein cholesterol; LDL-C, low density lipoprotein cholesterol; GLU, serum glucose; HGS, absolute handgrip strength; RHGS, relative handgrip strength. Absolute handgrip strength and relative handgrip strength were standardized before entering into the logistic regression model. Model 1: adjusted for age, smoking, alcohol drinking, and educational status; Model 2: Model 1+ adjusted for BMI.

**Figure 4:**
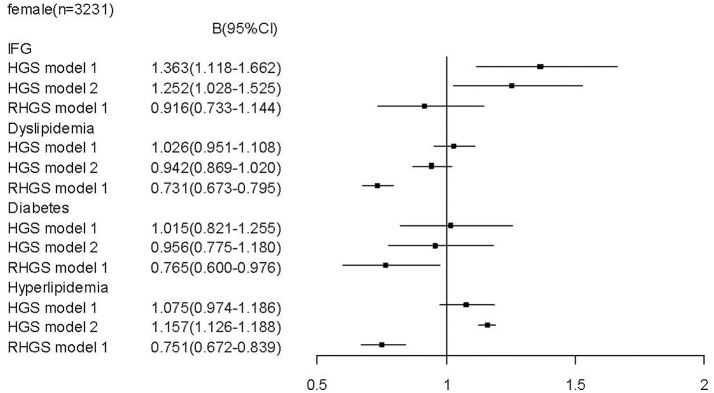
Association of absolute handgrip strength, relative handgrip strength with metabolic disease in female subjects.TC, total cholesterol; logTG, means of log-transformed for triglyceride; HDL-C, high density lipoprotein cholesterol; LDL-C, low density lipoprotein cholesterol; GLU, serum glucose; HGS, absolute handgrip strength; RHGS, relative handgrip strength. Absolute handgrip strength and relative handgrip strength were standardized before entering into the logistic regression model. Model 1: adjusted for age, smoking, alcohol drinking, and educational status; Model 2: Model 1+ adjusted for BMI.

To determine whether relative handgrip strength (that is corrected to BMI) and/or absolute handgrip strength are the reasonable predictors for metabolic profile and metabolic disease, we assessed the association of both parameters with the metabolic assessments. In males, we found that unlike a higher relative handgrip strength being associated or trended to be associated with a more favorable metabolic profile, a higher absolute handgrip strength was paradoxically associated with an unfavorable metabolic profile, including higher levels of LDL-C [*b* = 0.033 (CI: 0.001, 0.065)], TG [*b* = 0.030 (CI: 0.019, 0.040)], TC [*b* = 0.050 (CI: 0.011, 0.088)]; and lower levels of HDL-C [b = −0.023 (CI:−0.037, −0.010)]. Moreover, when these associations were adjusted for BMI (Figure 1), there remained this undesirable association to an unfavorable metabolic profile. Furthermore, in contrast the strong association of high relative handgrip strength to a prevalence of lower risk of preclinical and established stages of metabolic disease, the higher absolute handgrip strength was associated with higher risk of prevalence of metabolic disease. Even after adjusting for BMI (Figure 3), it still did not negate the association to preclinical and established stages of metabolic disease, in contrast to the relative handgrip strength assessment. The results from female subjects were similar to that from male subjects (Figures 2, 4).

## Discussion

In this study, we have demonstrated that relative handgrip strength was associated with a favorable metabolic profile and a lower risk of prevalence of preclinical and established stages of metabolic disease. Additionally, we found that relative handgrip strength was a more reasonable predictor of metabolic profile and metabolic disease than absolute handgrip strength. To the best of our knowledge, this study is the first to assess the association between relative handgrip strength and metabolic disease in general population; and also the first to compare the distinct differences between the association of absolute handgrip strength versus relative handgrip strength to metabolic profile and metabolic disease.

In this study, we found that higher relative handgrip strength was associated with lower risk of prevalence of hyperlipidemia and dyslipidemia in both genders. The rationale for this association may due to the higher relative handgrip strength was associated with the favorable profile of lipid metabolism (HDL-C, LDL-C, TG, TC) in this study population, which was consistent with Lee's study (Lee et al., 2016) and likely contributed to or accounted for the lower prevalence of dyslipidemia and hyperlipidemia.

We also demonstrated that higher relative handgrip strength was associated with lower risk of impaired fasting glucose in males, but not in females. This may be explained by the benefit effect of estrogen on female metabolism (Geer and Shen, 2009; Basu et al., 2017), which might overcoming or attenuating the adverse effect of lower relative handgrip strength on glucose metabolism.

In contrast to relative handgrip strength, we found that absolute handgrip strength measured alone was paradoxically associated with poorer metabolic profiles, preclinical and established stages of metabolic disease. This counter-intuitive and distinct association between absolute and relative handgrip strength could be attributed to the influence of body weight, whereby overweight and obese subjects had higher absolute handgrip strength. However, overweight or obesity are associated with unfavorable metabolic profile, and in this study overweight and obese participants accounted for 53% (*n* = 2,940) of the study population. So we speculated that overweight and obesity were the confounding factors which distorted the true association between absolute handgrip strength and metabolism. This could be supported by our results. After adjusted the association of absolute handgrip strength and metabolic profile and diseases by BMI, we demonstrated that almost all the unreasonable associations were corrected to a certain extent (Figures 1–4). This could be at least partially explained this counter-intuitive association. Some studies suggested obesity-related adverse metabolic effect mainly occured in subjects with upper body fat accumulation, the detrimental distribution, commonly associated with visceral obesity (Booth et al., 2014). Whereas BMI is only a measure to reflect the overall body fat composition, but not fat distribution. Therefore we infer if the association of absolute handgrip strength with metabolic profile and diseases was adjusted with the measure of fat distribution, such as visceral fat contents, the counter-intuitive association could be further reversed. Thus these results also indicated that relative handgrip strength is a more reasonable marker to predict metabolic profile and diseases.

Several limitations should be considered in this study. First, the cross-sectional design rendered it unable to demonstrate a causal relationship between relative handgrip strength and metabolic profile and metabolic disease. Second, although we had adjusted for age, sex, smoking, alcohol drinking, and educational level, we still cannot exclude other possible contributory factors that could have influenced the results, such as physical training, especially on the muscle group in the arm and the use of lipid-lowering drugs. Third, diseases were self-reported by the participants. Despite these limitations, our study has its strengths. We used a multi-stage randomized sampling method to obtain research subjects, which avoided selection bias and ensured that the sampling was representative of the general population in China.

## Conclusion

Higher relative handgrip strength was associated with a favorable metabolic profile and lower risk of preclinical and establishedstages of metabolic disease. Measurement of relative handgrip strength is a more reasonable predictor of metabolic profile and metabolic disease than the absolute handgrip strength measurement.

## Resource identification initiative

The SPSS software version 23 for Windows (SPSS Inc., Chicago, IL, USA, RRID:SCR_002865). R version 3.3.1(R development core team; available from http://www.r-project.org/, RRID:SCR_001905).

## Author contributions

DL and GG: Carried out the experimental design, participated in the data analysis, and drafted the manuscript. They contributed equally to this study and share first authorship. LX and BZ: Participated in the literature search, performed the data analysis, and reviewed the manuscript. FL, JM, ZH, and YL: Participated in the data collection and reviewed the manuscript. WL, JJ, XY, and HG: Participated in the experimental design and the review of the manuscript. GS and YH: Contributed to the experimental design and review of this manuscript. All authors read and approved the final manuscript.

### Conflict of interest statement

The authors declare that the research was conducted in the absence of any commercial or financial relationships that could be construed as a potential conflict of interest. The reviewer RRC and handling Editor declared their shared affiliation.
